# Prognostic value of temporal patterns of global longitudinal strain in patients with chronic heart failure

**DOI:** 10.3389/fcvm.2022.1087596

**Published:** 2023-01-12

**Authors:** Sabrina Abou Kamar, Yaar S. Aga, Marie de Bakker, Victor J. van den Berg, Mihai Strachinaru, Dan Bowen, René Frowijn, K. Martijn Akkerhuis, Jasper Brugts, Olivier Manintveld, Victor Umans, Marcel L. Geleijnse, Eric Boersma, Bas M. van Dalen, Isabella Kardys

**Affiliations:** ^1^Department of Cardiology, Erasmus University Medical Center, Rotterdam, Netherlands; ^2^Netherlands Heart Institute, Utrecht, Netherlands; ^3^Department of Cardiology, Franciscus Gasthuis and Vlietland, Rotterdam, Netherlands; ^4^Department of Cardiology, Northwest Clinics, Alkmaar, Netherlands; ^5^Department of Anesthesiology, Leiden University Medical Center, Leiden, Netherlands

**Keywords:** global longitudinal strain, left ventricle ejection fraction, heart failure, repeated measurements, longitudinal studies, NT-proBNP

## Abstract

**Background:**

We investigated whether repeatedly measured global longitudinal strain (GLS) has incremental prognostic value over repeatedly measured left ventricular ejection fraction (LVEF) and N-terminal pro B-type natriuretic peptide (NT-proBNP), and a single “baseline” GLS value, in chronic heart failure (HF) patients.

**Methods:**

In this prospective observational study, echocardiography was performed in 173 clinically stable chronic HF patients every six months during follow up. During a median follow-up of 2.7 years, a median of 3 (25th–75th percentile:2–4) echocardiograms were obtained per patient. The endpoint was a composite of HF hospitalization, left ventricular assist device, heart transplantation, cardiovascular death. We compared hazard ratios (HRs) for the endpoint from Cox models (used to analyze the first available GLS measurements) with HRs from joint models (which links repeated measurements to the time-to-event data).

**Results:**

Mean age was 58 ± 11 years, 76% were men, 81% were in New York Heart Association functional class I/II, and all had LVEF < 50% (mean ± SD: 27 ± 9%). The endpoint was reached by 53 patients. GLS was persistently decreased over time in patients with the endpoint. However, temporal GLS trajectories did not further diverge in patients with versus without the endpoint and remained stable during follow-up. Both single measurements and temporal trajectories of GLS were significantly associated with the endpoint [HR per SD change (95%CI): 2.15(1.34–3.46), 3.54 (2.01–6.20)]. In a multivariable model, repeatedly measured GLS maintained its prognostic value while repeatedly measured LVEF did not [HR per SD change (95%CI): GLS:4.38 (1.49–14.70), LVEF:1.14 (0.41–3.23)]. The association disappeared when correcting for repeatedly measured NT-proBNP.

**Conclusion:**

Temporal evolution of GLS was associated with adverse events, independent of LVEF but not independent of NT-proBNP. Since GLS showed decreased but stable values in patients with adverse prognosis, single measurements of GLS provide sufficient information for determining prognosis in clinical practice compared to repeated measurements, and temporal GLS patterns do not add prognostic information to NT-proBNP.

## Introduction

Left ventricular ejection fraction (LVEF) is the most commonly used parameter to evaluate LV systolic function in chronic heart failure (HF) patients. The use of LVEF in chronic HF patients carries several known limitations, such as high variability, geometric assumptions, load dependency, and a low reproducibility (1, 2). Furthermore, previous studies have shown that the predictive value of LVEF for cardiac events in HF patients leaves room for improvement (1, 3, 4).

Global longitudinal strain (GLS) is independently associated with all-cause mortality, cardiovascular death and heart transplantation (2, 5, 6), and also predicts risk of HF hospitalization (4, 7). When compared to LVEF, GLS has incremental prognostic value (3, 8, 9) and carries potential to be used as a standard measurement for chronic HF (10). However, most of the studies on GLS in HF have focused on a single measurement of GLS, which only reflects a snapshot of the patient’s physiological state. The prognostic value of repeated measurements of GLS in chronic HF patients has not been addressed and has never been compared with repeatedly measured LVEF.

Therefore, we hypothesize that temporal patterns of GLS are associated with adverse clinical events in chronic HF patients, and that temporal patterns of GLS may provide incremental value to temporal patterns of LVEF and N-terminal pro-brain natriuretic peptide (NT-proBNP, since this is the blood biomarker most commonly used for prognostication in HF). To test this hypothesis, we repeatedly measured GLS, LVEF, and NT-proBNP in 173 clinically stable patients with chronic HF. Moreover, we compared the prognostic value of repeatedly measured GLS with a single “baseline” GLS value.

## Materials and methods

### Study design

Details on the design of the Serial Biomarker Measurements and New Echocardiographic Techniques in Chronic Heart Failure Patients Result in Tailored Prediction of Prognosis (Bio-SHiFT) study have been published previously (11). In short, Bio-SHiFT is a prospective, observational cohort of stable patients with chronic HF, conducted in the Erasmus MC, Rotterdam, and Northwest clinics, Alkmaar, The Netherlands. Patients were recruited during their regular outpatient visits while in clinically stable condition (i.e., they had not been hospitalized for HF in the 3 months prior to inclusion). The main inclusion criteria were diagnosis of HF 3 or more months before inclusion according to the then prevailing guidelines of the European Society of Cardiology (12), and age ≥ 18 years. Patients were followed for a maximum of 30 months, during which study follow-up visits were scheduled every 3 months. At each visit, a short medical evaluation was performed, and blood samples were drawn. During the study, the routine outpatient follow-up by the treating physician continued for all patients, independently of the study visits. The study was approved by the medical ethics committees, conducted in accordance with the Declaration of Helsinki, and registered in ClinicalTrials.gov (NCT01851538). All patients signed informed consent for the study. A total of 398 patients were included in Bio-SHiFT. All patients included at Erasmus MC were eligible for the repeated echo substudy. This substudy included a total of 175 patients in whom echocardiography was performed every 6 months during follow-up (13). Two patients had insufficient image quality. The remaining 173 patients were included in the analysis.

### Echocardiography measurements and evaluation

Two-dimensional gray-scale harmonic images were obtained in the left lateral decubitus position. Standard apical four-, three-, and two-chamber views were recorded. A commercially available ultrasound system was used (iE33, Philips, Best, Netherlands), equipped with a broadband (1–5 MHz) S5-1 transducer (frequency transmitted 1.7 MHz, received 3.4 MHz). Images were stored in the echo core lab of Erasmus MC (13). Using specialized software (2D Cardiac Performance Analysis version 4.5; TomTec Imaging Systems, Unterschleissheim, Germany), LVEF, end-diastolic and end-systolic LV diameter, and end-systolic left atrial diameter were measured. The vena cava inferior diameter, the tricuspid regurgitation (TR) velocity, and the function of the mitral, and tricuspid valves were also assessed. The diastolic parameters were evaluated using Philips Excellera version R4.1 (Philips Medical Systems, Netherlands) or TomTec Imaging Systems. To assess diastolic function, the peak early filling velocity (E)/late filling velocity (A) ratio and the ratio of the E and early diastolic mitral annular velocity (e’) were calculated. For the e’, we used the mean of the lateral and medial e’ when available; however, if only one of the two was available, this value was used (14). All echocardiographic measurements were performed blinded to biomarker and clinical event data.

Strain analysis based on speckle tracking echocardiography was also performed using TomTec Imaging Systems. A frame rate above 30 f/s is sufficient for accurate GLS assessment (15), and all the echoes had a frame rate of 30 f/s or higher; with the majority of the echoes having a frame rate of 50 f/s, and a part of the echoes performed in the beginning of the study having a rate of 30 f/s. The images were analyzed retrospectively after completion of follow-up by a single operator, who was blinded to other echocardiographic parameters and the patients’ characteristics. The GLS assessment of the left ventricle was performed in 18 LV segments on the standard apical four-, three-, and two-chamber views, where the endocardial border was traced manually at end systole. We only obtained GLS if tracking was sufficient in ≥5 of the 6 segments per view. Extremely low values of GLS (<−5%) were verified by a second observer. If a patient had AF during the echocardiography, the index beat method was used. This is a validated method to measure echocardiographic parameters during AF (16). The mean GLS from the three apical views was considered the LV GLS. By convention, GLS results were interpreted as absolute values (17). In other words, a change of GLS from for example −18 to −15% will be reported as a decrease of GLS. Intra-observer reproducibility was assessed by re-measuring GLS in 20 echoes and calculating the intraclass correlation coefficient.

### NT-proBNP measurement

During each study visit (every 3 months), blood samples were drawn to measure a set of biomarkers, including NT-proBNP. Blood samples were processed and stored at −80°C within 2 h after collection. To determine NT-proBNP levels, a batch analysis was performed using an electrochemiluminescence immunoassay (Elecsys 2010; Roche Diagnostics, Indianapolis, IN, USA). Accordingly, results of the biomarker assays were not available to treating physicians at the time of the outpatient visits and did not interfere with usual care.

### Clinical study endpoints

The primary endpoint comprised the composite of hospitalization for the management of acute or worsened HF, left ventricular assist device (LVAD) implantation, cardiac transplantation, and cardiovascular death, whichever occurred first in time. All events were adjudicated by a clinical event committee blinded to the echocardiographic assessments and biomarker measurements, after reviewing corresponding hospital records and discharge letters.

### Statistical analyses

Distributions of continuous variables were tested for normality using the Shapiro-Wilk test. Normally distributed continuous variables are presented as mean ± standard deviation (SD), and non-normally distributed variables as median and interquartile range (25th–75th percentile). Categorical variables are presented as numbers and percentages. Differences in baseline characteristics between patients who experienced the endpoint and those who did not were tested using the *t*-test and Mann–Whitney test, according to variable distributions, for continuous variables, and χ^2^-tests and Fisher’s exact tests, when appropriate, for categorical variables.

We evaluated the association of baseline clinical and echocardiographic characteristics with baseline GLS using linear regression, with GLS being the dependent variable. Moreover the Pearson correlation coefficient was calculated to examine the correlation between the variables of interest. Then we used linear mixed models to examine the associations of baseline clinical characteristics with repeatedly measured GLS, as well as the associations of repeatedly measured echocardiographic parameters and repeatedly measured GLS. Random effects were used to account for the presence of multiple echocardiograms per patient.

Hereafter, we assessed the value of repeated echocardiographic measurements for prediction of the endpoint, as well as their incremental value to sole, baseline measurements. We used the framework of joint models for longitudinal and survival data (18). In these joint models, a linear mixed effects (longitudinal) model provided estimates of the individual temporal trajectories of the echo parameters. These estimated trajectories were combined with a relative risk model, to study their association with the risk of the study endpoint. The individual trajectories were adjusted for all variables that showed statistically significant differences between patients with and without the endpoint (*p* < 0.05; age, sex, duration of HF, systolic blood pressure, diastolic blood pressure, renal failure, and atrial fibrillation). The associations between the temporal evolutions of GLS and the endpoint, resulting from the relative risk model, were first only adjusted for age and sex. Thereafter, baseline LVEF and baseline NT-proBNP levels were added consecutively. Lastly, all variables with significant differences between those with and without the endpoint were added. To investigate the incremental value of repeatedly measured GLS to repeatedly measured LVEF and NT-proBNP, we combined the repeated measurements of each of these variables in multivariable joint models.

To enable comparisons of effect sizes of different variables, prior to the analyses, all investigated echo parameters, and the NT-proBNP measurements, were first log transformed to achieve a normal distribution, after which the corresponding Z-scores were calculated. For GLS no transformation was needed. The first echoes were selected and entered into Cox models to obtain the hazard ratios (HRs) entailed by the first echoes only. To obtain the HRs entailed by the repeatedly measured echoes, joint models were used. Thus, the results of the regression analyses of the Cox and joint models can be directly compared and are presented as HRs, which represent risk per SD increase/decrease of the standardized variable, along with the corresponding 95% confidence interval (CI).

As described above, one of our aims was to investigate whether repeatedly measured GLS carries incremental predictive value to repeatedly measured LVEF and NT-proBNP. We chose to present our results solely as adjusted HRs and not to combine them with C-statistics. Pepe et al. (19) have demonstrated that testing for improvement in prediction performance is not necessary if a variable has already been shown to be an independent risk factor, and that standard testing procedures for C-indices are very conservative and thus insensitive to improvements in prediction performance.

Missing values in GLS and the other echo parameters were, except for the A wave, always due to poor image quality and were as such missing completely at random. Accordingly, we chose to perform a complete case analysis. Missing values for the A wave were mostly due to atrial fibrillation during the echo or due to mitral valve replacement or clipping. In this specific patient group imputation of missing values is inappropriate, as the A wave can never be measured. Thus, we again chose for a complete case analysis here. The results of this analysis should not be extrapolated to patients excluded from the analysis.

All analyses were performed with R Statistical Software using packages nlme (20) and JMbayes (18). All tests were two-tailed, and *p* values < 0.05 were considered statistically significant.

## Results

### Baseline characteristics and clinical endpoints

From October 2011 to January 2018, 173 patients were included. All patients had EF < 50%, with a mean ± SD LVEF of 27 ± 9%. In 150 patients, EF was below 40% (HFrEF). The remaining 23 patients had an EF between 40% and 49% (HFmrEF) (21). Mean age was 58 ± 11 years, 76% were men, and mean BMI was 27.6 ± 4.7 kg/m^2^. The median time between diagnosis of HF and inclusion in the study was 6.8(6.3–7.3) years. The highest proportion of the patients was in NYHA class II (55%) and 41% had HF due to ischemic heart disease. There was no significant difference in proportions of males and females between the patients who reached the endpoint and remained endpoint free (Table 1).

**TABLE 1:** Baseline patient characteristics in relation to the composite endpoint.

	Overall	Endpoint-free	Endpoint	*P*-Value
*N*	173	120	53	
**Demographics**				
Males, *n* (%)	132(76)	92(76)	40(75)	1
Age, years (mean(SD))	58.0(11.2)	57.3(11.4)	59.6(10.8)	0.2
**Clinical characteristics**				
Duration of HF, years [median (25th–75th percentile)]	6.8(6.3-7.3)	6.5(5.9-7.1)	8.1(7.0-9.2)	**<0**.**001**
Body mass index, kg/m^2^ (mean(SD))	27.5(4.7)	27.6(4.7)	27.2(4.5)	0.5
Heart rate, bpm (mean(SD))	67(12.9)	67(14.5)	67(8.5)	0.8
Systolic blood pressure, mmHg (mean(SD))	108(18.3)	110(18.4)	102(17.1)	**0**.**008**
Diastolic blood pressure, mmHg (mean(SD))	67(9.8)	68(9.8)	65(9.3)	**0**.**03**
NYHA class (%)				**0**.**009**
I	45(26.3)	39(33)	6(12)	
II	94(55)	62(52)	32(62)	
III	32(19)	18(15)	14(27)	
NT-proBNP, pmol/L [median (25th–75th percentile)]	118[31,223]	73[25,175]	235[140,410]	**<0**.**001**
**Features of HF**				
Ischemic heart disease (%)	71(41)	44(37)	27(51)	0.1
Hypertension (%)	2(1)	2(2)	0(0)	0.9
Cardiomyopathy (%)	73(42)	52(43)	21(40)	0.8
Secondary to valvular heart disease (%)	4(2)	2(2)	2(4)	0.8
Other etiology of HF (%)	16(28)	14(17)	2(1)	0.8
Unknown (%)	9(5)	8(7)	1(2)	0.4
**Medical history**				
Myocardial Infarction (%)	69(40)	43(36)	26(50)	0.1
PCI (%)	62(36)	43(36)	19(36)	1
CABG (%)	16(9)	10(8)	6(11)	0.7
Atrial fibrillation (%)	53(31)	28(23)	25(47)	**0**.**003**
Diabetes Mellitus (%)	40(23)	26(22)	14(26)	0.6
Chronic renal failure (%)	69(40)	38(32)	31(59)	**0**.**002**
COPD (%)	24(14)	15(13)	9(17)	0.6
**Medication use**				
Beta blockers (%)	165(95)	116(97)	49(93)	0.4
ACE inhibitors (%)	120(69)	84(70)	36(68)	0.9
Angiotensin II receptor blockers (%)	48(28)	34(28)	14(26)	0.9
Loop diuretics (%)	161(93)	108(90)	53(100)	**0**.**039**
Aldosteron antagonists (%)	128(74)	84(70)	44(83)	0.1

Bold values represent the statistically significant differences at *p* < 0.05.

In total, the composite endpoint was reached by 53 patients, and first occurrence of any of the components was as follows; 40 patients were re-hospitalized for acute or worsened HF, six patients received a heart transplantation, four patients received an LVAD implantation, and three patients died from cardiovascular causes. Patients who reached the composite endpoint had a significantly lower LVEF, longer duration of HF at study inclusion, lower systolic and diastolic blood pressure and higher NT-proBNP levels (Table 1).

### Echocardiography

During a median (25th–75th percentile) follow-up time of 2.7 (2.5–2.8) years, 505 echocardiograms were performed with a median (25th–75th percentile) of 3 (2–4) echoes per patient. Patients had up to eight consecutive echocardiographic evaluations performed with 65% of patients having at least three evaluations. Missing echocardiograms mostly occurred due to logistic circumstances (e.g., the unavailability of an ultrasound technician during the study visit). GLS was successfully measured in 96% of the total of 505 echocardiograms. Missing values were due to insufficient image quality (90% of these missing values of these missing values) or the absence of one of the apical views (10% of these missing values). The intraclass correlation coefficients for intra-observer reproducibility were 0.91 and 0.85 for GLS and LVEF, respectively.

### First available echocardiogram

The characteristics of the first available echocardiogram for each patient are presented in Table 2. Due to logistic reasons, 55% of these first available echoes were performed at baseline (follow-up time zero), 12.8% of the first available echoes were performed during the first follow-up visit (target follow-up time 3 months), 18% during the second follow-up visit (target 6 months), and the remaining 14.2% thereafter. The date of the first available echocardiogram was considered as the start of follow-up. After the first available echoes, subsequent echocardiograms were performed every six months during follow-up (Supplementary Figure 1).

**TABLE 2:** Echocardiographic characteristics from first available echo in relation to the composite endpoint.

	Endpoint-free	Endpoint	*P*-Value	Missing values
**Systolic parameters**				
LV GLS,% [mean (SD)]	−10.1(3.6)	−6.4(2.3)	**<0**.**001**	13(8%)
LVEF,% [mean (SD)]	31.1(9.8)	22.9(9.2)	**<0**.**001**	10(6%)
Systolic LV diameter, mm (median[25th–75th percentile])	53.00[46.3,62.0]	60.00 [54.0, 70.5]	**<0**.**001**	16 (9%)
Systolic LA diameter, mm [mean(SD)]	40.3(7.6)	48.3(7.5)	**<0**.**001**	18(10%)
TR velocity, m/s (median[25th–75th percentile])	2.40[2.03,2.65]	2.62[2.29,3.03]	**0**.**04**	56(32%)
**Diastolic parameters**				
Left atrial volume index, ml/m^2^ [mean(SD)]	34.5(5.3)	49.2(5.8)	**<0**.**001**	18(10%)
E/A ratio [mean(SD)]	1.17(0.88)	2.19(1.05)	**<0**.**001**	44(25%)*
E/e’ ratio [mean(SD)]	12.9(7.3)	21.4(10.2)	**<0**.**001**	20(12%)
Diastolic LV diameter, mm (median[25th–75th percentile])	63.0[57.0,70.0]	67.0[63.0,77.0]	**0**.**003**	14(8%)
**Vena Cava**				
Inferior vena cava, mm(median[25th-75th percentile])	14.70[12.00,17.50]	20.00[16.00,23.55]	**<0**.**001**	39(23%)
VCI sniff test: No (%)	4(4)	14(35)	**<0**.**001**	43(25%)
**Heart valve diseases**				
Mitral valve regurgitation (%)			**<0**.**001**	13(8%)
None	47(42)	6(13)		
Mild	43(38)	29(60)		
Moderate	20(18)	7(15)		
Severe	2(2)	6(13)		
Tricuspid valve regurgitation (%)			**<0**.**001**	16(9%)
None	68(61)	16(35)		
Mild	36(32)	18(39)		
Moderate	6(5)	6(13)		
Severe	1(1)	6(13)		
Aortic valve stenosis (%)			0.1	16(9%)
None	109(99)	44(94)		
Mild	1(1)	2(4)		
Moderate	0(0)	1(2)		
Aortic valve regurgitation (%)			**0.01**	16(9%)
None	101(92)	36(77)		
Mild	8(7)	7(15)		
Moderate	1(1)	4(9)		

*Missing values due to atrial fibrillation during the echo or due to mitral valve replacement or clipping. Bold values represent the statistically significant differences at *p* < 0.05.

Patients who reached the composite endpoint had a significantly decreased GLS with a mean difference of 3.7% (95%CI: 2.6–4.7) and a lower LVEF with a mean difference of −9% (95%CI: −12.00,−5.88) compared to patients who remained endpoint-free (Table 2). The dimensions of the left ventricle, left atrium, and inferior vena cava were significantly larger than those of patients who did not reach the endpoint. Moreover, patients who reached the endpoint had higher E/A ratio, E/e’ ratio and TR velocities (Table 2).

### Associations of baseline and serially measured GLS with clinical and echocardiographic characteristics

**FIGURE 1:**
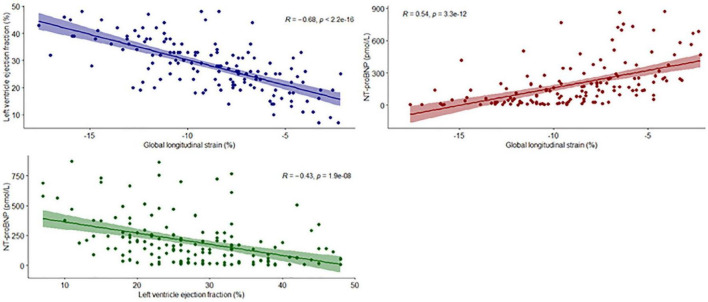
Scatterplots for GLS, LVEF and NT-proBNP. The regression lines represent the correlation between the variables of interest. Each dot represents a single patient.

Supplementary Tables 1, 2 display the associations of baseline GLS with clinical and echocardiographic characteristics. GLS showed the strongest association with LVEF and was significantly decreased in patients in a higher NYHA class and patients with other comorbidities, indicating worse LV function. Although GLS was decreased in men compared with women, this did not translate into a higher incidence of PEP in men (Table 1). GLS was also significantly different between patients with and without ischemic HF, with a mean (95%CI) of −7.7%(−8.5 to −6.9%) and −9.9%(−10.6 to −9.1%) respectively. Baseline GLS and LVEF showed a moderate to strong correlation (*r* = −0.68, *p* < 0.001), which was stronger than the correlation between GLS and NT-proBNP (*r* = 0.54, *p* < 0.001). Scatterplots are depicted in Figure 1.

**FIGURE 2:**
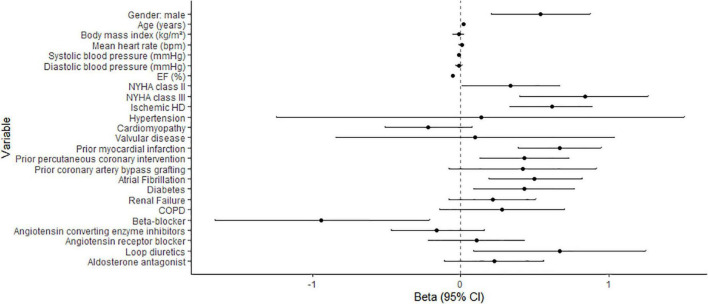
Association of baseline clinical characteristics with serially measured GLS. Betas depict the change in GLS (in%) when the explanatory variable is increased by 1 unit. 95%CI: 95% confidence interval.

Associations remained essentially the same when examined for longitudinally measured GLS (Figure 2 and Supplementary Table 3). GLS showed significant associations with almost all examined echocardiographic parameters. The strongest association was found with the E/A ratio and TR velocities. Repeatedly measured echocardiographic parameters also remained strongly associated with repeatedly measured GLS (Supplementary Figure 3 and Supplementary Table 4).

### Baseline and repeatedly measured GLS in relation to the composite endpoint

When entered into separate models, baseline GLS and LVEF were both significantly associated with the endpoint, independently of age, sex, baseline NT-proBNP and the duration of HF (Table 3), with HRs(95%CI) per SD change of 2.15(1.34–3.46) and 1.41(1.01–2.13), respectively. When entered into one model corrected for the same covariates, the association of baseline GLS with the endpoint remained [HR(95%CI: 2.76(1.66–4.58)], while that of LVEF disappeared [HR(95%CI: 1.11(0.71–1.75)]. GLS was also significantly associated with the endpoint after adjustment for the most important systolic and diastolic echocardiographic parameters, namely EF, E/A ratio, E/e’ and LAVI [HR(95%CI: 1.75(1.30–2.85)].

**TABLE 3:** Associations of the baseline and repeatedly measured GLS with the primary endpoint.

	HR (95%CI)	*P*-Value
**Baseline measurements**		
GLS*	2.15(1.34–3.46)	<0.001
LVEF*	1.41(1.01–2.13)	0.04
GLS and LVEF*		
GLS	2.76(1.66–4.58)	<0.001
LVEF	1.11(0.71–1.75)	0.6
GLS and NT-proBNP**		
GLS	2.15(1.34–3.46)	0.002
NT-proBNP	1.82(1.07–3.09)	0.03
**Repeated measurements of GLS**		
Model 1	3.33(1.95–6.09)	<0.001
Model 2	3.54(2.01– 6.20)	<0.001
Model 3	3.50(2.18–5.89)	<0.001
Model 4	1.75(1.30–2.85)	<0.001
Model 5	4.04(2.34–7.40)	<0.001
**Repeated measurements of GLS and LVEF or NT-proBNP**		
Model 6		
GLS	4.38(1.49–14.70)	0.008
LVEF	1.14(0.41–3.23)	0.8
Model 7		
GLS	0.79(0.47–1.30)	0.4
NT-proBNP	2.90(1.59–5.55)	<0.001

*Corrected for age, sex, baseline NT-proBNP and HF duration.

**Corrected for age, sex, HF duration.

Model 1: corrected for age, sex, HF duration, baseline LVEF.

Model 2: corrected for age, sex, HF duration, baseline NT-proBNP.

Model 3: corrected for age, sex, HF duration, baseline LVEF and NT-proBNP.

Model 4: corrected for age, sex, HF duration, baseline LVEF, E/A ratio, LAVI.

Model 5: corrected for age, sex, HF duration, New York Heart Association (dichotomized as NYHA class I-II versus NYHA class III-IV), atrial fibrillation, renal failure, systolic and diastolic blood pressure.

Model 6 and 7: multivariable Joint Models: Corrected for age, sex, HF duration, New York Heart Association (dichotomized as NYHA class I-II versus NYHA class III-IV), atrial fibrillation, renal failure, systolic and diastolic blood pressure.

**FIGURE 3:**
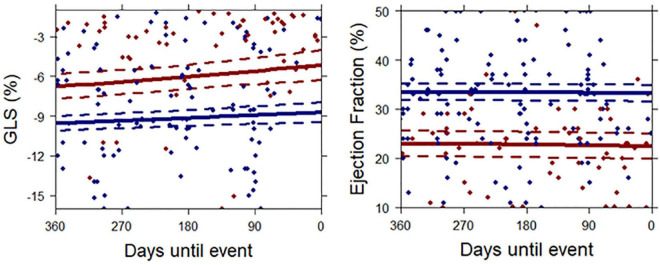
Mean temporal patterns of GLS and LVEF until occurrence of the primary endpoint or censoring. Continuous lines represent mean temporal patterns for patients with the endpoint (red) and patients who remained endpoint-free (blue), as extracted from the joint model. Time-point zero represents the occurrence of an event in the endpoint patients and censoring in patients who remained endpoint-free. Dotted lines represent 95% confidence intervals. Each dot represents a single measurement.

In the total population, there was a slight decrease in GLS over time as the endpoint or censoring approached (Beta[95%CI]: 0.71[0.47–0.94] per SD change of GLS per year), *p* < 0.001). Figure 3 and Supplementary Figure 3 show the temporal evolution of GLS in patients who experienced the endpoint and those who did not. At 12 months before the endpoint or censoring occurred, GLS was already decreased in patients that later experienced the endpoint compared to those who did not; and it remained decreased as the endpoint approached. However, the curves were parallel for patients with and without the endpoint, with no significant difference in slope, similar to the temporal evolution of LVEF (Figure 3 and Supplementary Figures 3, 5). The temporal evolution of E/A and E/e’ (Supplementary Figure 4) also showed similar patterns. When we calculated, from the mixed models, the mean relative change in GLS and LVEF compared to baseline values of GLS and LVEF in the patients with the endpoint, on average, for GLS, these patients had a relative decrease of 5% compared to baseline at day 185. This was 255 days earlier than the relative decrease of LVEF of 5% at day 440.

Accordingly, longitudinally measured GLS was significantly associated with the endpoint in all the fitted joint models (Table 3). In the first model, adjusted for age, sex and duration of HF, the HR was 2.11 (95%CI: 1.37–3.31). When baseline LVEF was added to the model, the HR was 3.33 (95%CI: 1.95–3.31). After adding baseline NT-proBNP to the models, the association still persisted [3.50(95%CI: 2.18–5.89)].

The results of the multivariable joint models into which the repeatedly measured GLS, as well as repeatedly measured LVEF and NT-proBNP, were entered, are shown in Table 3. GLS showed a HR(95%CI) of 4.38(1.49–14.70) for the endpoint when correcting for repeatedly measured LVEF. However, the HR(95%CI) became 0.79(0.47–1.30) when correcting for repeatedly measured NT-proBNP.

## Discussion

In this study consisting of 173 chronic HF patients with reduced EF, that had limited symptoms at baseline, firstly, temporal evolution of GLS was significantly associated with adverse cardiovascular events during a median follow-up of 2.7 years, independent of both baseline and repeated LVEF and baseline NT-proBNP measurements. However, the association disappeared after adjustment for repeated NT-proBNP measurements. Secondly, while GLS was decreased in patients that later experienced the endpoint as compared to those who did not, and remained decreased as the endpoint approached, the temporal trajectories of GLS did not further diverge in patients with versus without the endpoint and remained stable over this 2.7-year time frame. For this reason, we infer that repeatedly measuring GLS over a short time frame does not provide additional incremental prognostic information over a single measurement, and a single baseline measurement of GLS provides sufficient information for prognostication in clinical practice.

Previously, we have examined the prognostic value of repeated measurements of LVEF, as well as repeated measurements of established diastolic echo parameters, in the context of the Bio-SHiFT study (13). Similar to GLS, the temporal trajectories of LVEF and the diastolic parameters did not diverge between patients with and without the endpoint. However, to our knowledge, the prognostic value of repeatedly measured GLS, and its added value over a single ‘baseline’ GLS assessment, and over repeatedly measured LVEF, has not yet been examined in patients with HF. Herewith, this study confirms and increases previous evidence on the added prognostic value of GLS over LVEF. Several studies have shown that “baseline” GLS carries prognostic value over LVEF (1, 3, 5, 7). A meta-analysis by Kamal et al. (3) showed that baseline GLS was more strongly associated with mortality than LVEF. In a study by Bertini et al. (22) in 1060 HF patients, baseline GLS showed incremental value over LVEF as well. These studies only examined baseline measurements of GLS, whereas our study contained multiple GLS measurements per patient.

Baseline GLS can be considered low in this cohort (mean[95%CI]: −9.2%[−9.5 to −8.8%]) compared to healthy populations. However, this is inherent to the study population, and studies in other HFrEF cohorts have shown similarly low GLS values (10). While in our study the association of repeatedly measured GLS with the endpoint persisted when correcting for repeatedly measured LVEF, it disappeared when correcting for repeatedly measured NT-proBNP. In advanced stages of HF, further reduction in already low values of GLS and LVEF is unlikely, whereas NT-proBNP may further increase in advanced HF stages. This may have contributed to the finding that the incremental value of GLS disappeared after correcting for repeatedly measured NT-proBNP. Furthermore, GLS and EF provide no information about the detrimental impact of LV dysfunction on the right ventricle, whereas NT-proBNP does. In addition, the presence of mitral regurgitation (MR) could negatively affect the validity of LVEF (23). In contrast, NT-proBNP has been shown to be a reliable biomarker in MR and is an independent predictor in this group (24). Previous studies have already shown that NT-proBNP carries strong prognostic value in HF (25, 26). Our study demonstrates that the prognostic value of NT-proBNP is independent of repeated GLS measurements, but not vice versa. Herewith, and in combination with the availability and ease of implementation of simple laboratory tests, our study further supports the use of NT-proBNP for prognostication in HF. It should be noted though, that NT-proBNP levels could be impacted due to the presence of AF, and that the NT-proBNP to BNP ratio varies according to heart rhythm (27). This should be taken into account when interpreting NT-proBNP levels in patients with AF. Prevalence of AF was higher among patients who reached the endpoint. To account for potential confounding, we adjusted the models for AF.

The use of GLS in clinical practice is currently limited due to inter-vendor variability, poor predictive ability in images with low quality and load-dependency (3, 28). Nevertheless, in 2015 a EACVI/ASE/Industry Task Force consensus document was published to standardize deformation imaging (29). Furthermore, GLS is known to have better intra-observer and inter-observer variability than LVEF (3). Also, several studies have shown the prognostic incremental value of GLS over LVEF when EF was normal. A meta-analysis which included 5,721 patients demonstrated that impaired GLS was present in patients with normal LVEF, and predicted cardiac events (3), which is also shown in another study (9). These studies show that LVEF also carries potential limitations regarding diagnosis and prognostication of HF and illustrate the potential incremental value of GLS over LVEF for prognostication in clinical practice.

Several limitations of our study warrant consideration. First, treating physicians were not blinded to the echocardiograms. However, GLS values were measured retrospectively and were not available for the physicians. Second, the number of endpoints in the study was limited, and consequently so was the number of variables that could be entered into the models. To prevent overfitting, we fitted multiple multivariable models containing different confounders, instead of one model containing all covariates. Although residual confounding might be present, all these models were corrected for NT-proBNP. In addition, we also corrected for the duration of HF, to control for possible lead time or length time bias. Furthermore, multicollinearity (highly correlated variables) could be present when GLS and LVEF are entered in the model (*r* = −0.68), so results should be interpreted with caution. Nevertheless, examining the prognostic value of GLS over LVEF warranted inclusion of both variables in the model. Finally, the patients in this echo substudy were relatively young and there was a high proportion of patients in NYHA classes I and II. Older patients with worse condition may have been less likely to participate in the substudy. However, prognosis of patients in advanced stages of HF is already known to be poor, while in a population like ours, differentiating between patients who reach an event and patients who remain event free remains more difficult. Therefore, parameters with high prognostic value are essential in this group particularly.

Altogether, in a population of chronic HF patients, temporal evolution of GLS was significantly associated with adverse cardiovascular events during a median follow-up of 2.7 years, independent of both baseline and repeated LVEF, and baseline NT-proBNP measurements. After correction for repeated NT-proBNP in a multivariable model, the association disappeared. We conclude that repeatedly measuring GLS over a short time frame does not seem to provide additional incremental prognostic information over a single measurement in clinical practice. The incremental prognostic value of repeatedly measured NT-proBNP over GLS, supports the use of NT-proBNP for prognostication in clinical practice. Further studies in larger and more diverse cohorts are needed to confirm our findings; moreover, use of temporal trajectories of GLS for other purposes, such as assessment of response to therapy, warrants further research.

## Data availability statement

The datasets presented in this article are not readily available because anonymized data that support the findings of this study will be made available to other researchers for purposes of reproducing the results upon reasonable request and in accordance with a data-sharing agreement. Requests to access the datasets should be directed to IK, i.kardys@erasmusmc.nl.

## Ethics statement

The studies involving human participants were reviewed and approved by Medical Ethics Committees of Erasmus MC. The patients/participants provided their written informed consent to participate in this study.

## Author contributions

SA: acquisition, analysis, and interpretation of data and writing the manuscript. YA, VB, MS, RF, JB, and OM: acquisition and interpretation of data and revising the manuscript. MB: analysis and interpretation of data and revising the manuscript. DB: interpretation of data and revising the manuscript. KA, VU, and MG: conception and design of the work, the acquisition and interpretation of data, and revising the manuscript. EB and BD: conception and design of the work, interpretation of data, revising the manuscript, and handling funding. IK: conception and design of the work, the acquisition, analysis and interpretation of data, revising the manuscript, and handling funding and supervision. All authors contributed to the article and approved the submitted version.
