# Supplementary material for: Prognostic value of temporal patterns of global longitudinal strain in patients with chronic heart failure
Source: Front Cardiovasc Med. 2023 Jan 12;9:1087596. doi: 10.3389/fcvm.2022.1087596 (PMC9878393; doi:10.3389/fcvm.2022.1087596)
Supplement: Supplementary file 1 [file Table_1.DOCX]

## Supplementary tables

Supplementary table 1: Association baseline GLS with clinical characteristics*

|  | β (95% CI) | P value |
| --- | --- | --- |
| Demographics |  |  |
| Gender: male | 0.44 (0.07 – 0.81) | 0.02 |
| Age (years) | 0.01 (-0.58 – 0.06) | 0.09 |
| Clinical characteristics |  |  |
| Body mass index (kg/m²) | -0.03 (-0.07 – 0.01) | 0.1 |
| Mean heart rate (bpm) | 0.01 (-0.01 – 0.02) | 0.2 |
| Systolic blood pressure (mmHg) | -0.02 (-0.03 – -0.01) | <0.001 |
| Diastolic blood pressure (mmHg) | -0.02 (-0.03 – -0.01) | 0.03 |
| NYHA class I (reference group) |  |  |
| NYHA class II | 0.38 (0.02 – 0.74) | 0.04 |
| NYHA class III | 0.91 (0.44 – 1.39) | <0.001 |
| Features of HF |  |  |
| Ischemic HD | 0.56 (0.25 – 0.87) | <0.001 |
| Hypertension | -0.04 (-1.45 – 1.40) | 1 |
| Cardiomyopathy | -0.26 (-0.58 – 0.06) | 0.1 |
| Valvular disease | -0.19 (-1.19 – 0.82) | 0.7 |
| Medical history |  |  |
| Prior myocardial infarction | 0.60 (0.30 – 0.91) | <0.001 |
| Prior percutaneous coronary intervention | 0.37 (0.05 – 0.70) | 0.03 |
| Prior coronary artery bypass grafting | 0.35 (-0.17 – 0.87) | 0.2 |
| Atrial Fibrillation | 0.35 (0.01 – 0.69) | 0.04 |
| Diabetes | 0.34 (-0.35 – 0.71) | 0.08 |
| Renal Failure | 0.19 (-0.14 – 0.51) | 0.3 |
| COPD | 0.23 (-0.21 – 0.67) | 0.3 |
| Medication use |  |  |
| Beta-blocker | -0.97 (-1.71 – -0.22) | 0.01 |
| Angiotensin converting enzyme inhibitors | -0.19 (-0.53 – 0.16) | 0.3 |
| Angiotensin receptor blocker | 0.14 (-0.22 – 0.49) | 0.5 |
| Loop diuretics | 0.81 (0.19 – 1.41) | 0.01 |
| Aldosterone antagonist | 0.39 (0.04 – 0.74) | 0.03 |

*The betas represent the mean change in GLS (in %) when the explanatory variable is increased by one unit, or the mean difference between two groups when the explanatory variable is categorical.

Supplementary table 2: Association baseline GLS with echocardiographic parameters*

|  | β (95% CI) | P value |
| --- | --- | --- |
| Systolic parameters |  |  |
| Ejection fraction (%) | -0.07 (-0.08 - -0.05) | <0.001 |
| Diastolic LV diameter (mm) | 0.01 (-0.01 – 0.02) | 0.5 |
| Systolic LV diameter (mm) | 0.05 (0.03 – 0.06) | <0.001 |
| Systolic Left Atrial diameter (mm) | 0.05 (0.03 – 0.06) | <0.001 |
| Diastolic parameters |  |  |
| E/A ratio | 0.33 (0.17 – 0.48) | <0.001 |
| E/e ratio | 0.05 (0.04 – 0.07) | <0.001 |
| TR velocity | 0.38 (0.11 – 0.66) | 0.006 |
| Vena Cava |  |  |
| Vena Cava Inferior | 0.06 (0.03 – 0.09) | <0.001 |
| Vena cava Sniff: No | 0.88 (0.38 – 1.38) | <0.001 |
| Mitral valve regurgitation |  |  |
| Mild | 0.62 (0.27 – 0.96) | <0.001 |
| Moderate | 0.69 (0.26 – 1.13) | 0.002 |
| Severe | 0.95 (0.25 – 1.65) | 0.008 |
| Tricuspid valve regurgitation |  |  |
| Mild | 0.33 (-0.01 – 0.66) | 0.05 |
| Moderate | 1.22 (0.66 – 1.79) | <0.001 |
| Severe | 0.80 (0.08 – 1.52) | 0.03 |

*The betas represent the mean change in GLS (in %) when the explanatory variable is increased by one unit, or the mean difference between two groups when the explanatory variable is categorical.

Supplementary table 3: Association serially measured GLS with clinical characteristics

|  | β (95% CI) | P value |
| --- | --- | --- |
| Demographics |  |  |
| Gender: male | 0.54 (0.21 – 0.88) | 0.002 |
| Age (years) | 0.02 (0.01 – 0.03) | 0.006 |
| Clinical characteristics |  |  |
| Body mass index (kg/m²) | -0.01 (-0.05 – 0.02) | 0.5 |
| Mean heart rate (bpm) | 0.01 (-0.01 – 0.02) | 0.2 |
| Systolic blood pressure (mmHg) | -0.01 ( -0.02 – 0.01) | 0.009 |
| Diastolic blood pressure (mmHg) | -0.01 (-0.03 – 0.01) | 0.1 |
| NYHA class I (reference group) |  |  |
| NYHA class II | 0.34 (0.01 – 0.67) | 0.04 |
| NYHA class III | 0.84 (0.40 – 1.27) | <0.001 |
| Features of HF |  |  |
| Ischemic HD | 0.62 (0.33 – 0.89) | <0.001 |
| Hypertension | 0.14 (-1.24 – 1.51) | 0.8 |
| Cardiomyopathy | -0.22 (-0.51 – 0.08) | 0.1 |
| Valvular disease | 0.10 (-0.84 – 1.04) | 0.8 |
| Medical history |  |  |
| Prior myocardial infarction | 0.67 (0.39 – 0.95) | <0.001 |
| Prior percutaneous coronary intervention | 0.43 (0.13 – 0.73) | 0.005 |
| Prior coronary artery bypass grafting | 0.42 (-0.08 – 0.92) | 0.1 |
| Atrial Fibrillation | 0.50 (0.19 – 0.82) | 0.002 |
| Diabetes | 0.43 (0.09 – 0.77) | 0.01 |
| Renal Failure | 0.22 (-0.08 – 0.51) | 0.2 |
| COPD | 0.28 (-0.14 – 0.70) | 0.2 |
| Medication use |  |  |
| Beta-blocker | -0.94 (-1.66 – -0.21) | 0.01 |
| Angiotensin converting enzyme inhibitors | -0.16 (-0.47 – 0.16) | 0.3 |
| Angiotensin receptor blocker | 0.11 (-0.22 – 0.43) | 0.5 |
| Loop diuretics | 0.67 (0.09 – 1.25) | 0.02 |
| Aldosterone antagonist | 0.23 (-0.11 – 0.56) | 0.2 |

*The betas represent the mean change in GLS (in %) when the explanatory variable is increased by one unit, or the mean difference between two groups when the explanatory variable is categorical.

Supplementary table 4: Association serially measured GLS with serially measured echocardiographic parameters

|  | β (95% CI) | P value |
| --- | --- | --- |
| Systolic parameters |  |  |
| Ejection fraction (%) | -0.05 (-0.06 – -0.04) | <0.001 |
| Diastolic LV diameter (mm) | 0.01 (-0.09 – 0.01) | 0.5 |
| Systolic LV diameter (mm) | 0.03 (0.02 – 0.04) | <0.001 |
| Systolic Left Atrial diameter (mm) | 0.03 (0.02 – 0.04 | <0.001 |
| Diastolic parameters |  |  |
| E/A ratio | 0.07 (0.01 - 0.13) | 0.02 |
| E/e ratio | 0.02 (0.01 - 0.03) | <0.001 |
| TR velocity | 0.18 (0.06 - 0.29) | 0.004 |
| Vena Cava |  |  |
| Vena cava inferior | 0.02 (0.01 - 0.04) | 0.008 |
| Vena cava Sniff: No | 0.31 (0.07 - 0.55) | 0.01 |
| Mitral valve regurgitation |  |  |
| Mild | 0.13 (-0.02 – 0.28) | 0.08 |
| Moderate | 0.35 (0.11 – 0.58) | 0.004 |
| Severe | 0.62 (0.26 – 0.97) | 0.008 |
| Tricuspid valve regurgitation |  |  |
| Mild | 0.24 (0.11 – 0.37) | <0.001 |
| Moderate | 0.59 (0.32 – 0.86) | <0.001 |
| Severe | 0.20 (-0.23 – 0.64) | 0.4 |

*The betas represent the mean change in GLS (in %) when the explanatory variable is increased by one unit, or the difference between two groups when the explanatory variable is categorical.

## Supplementary figures


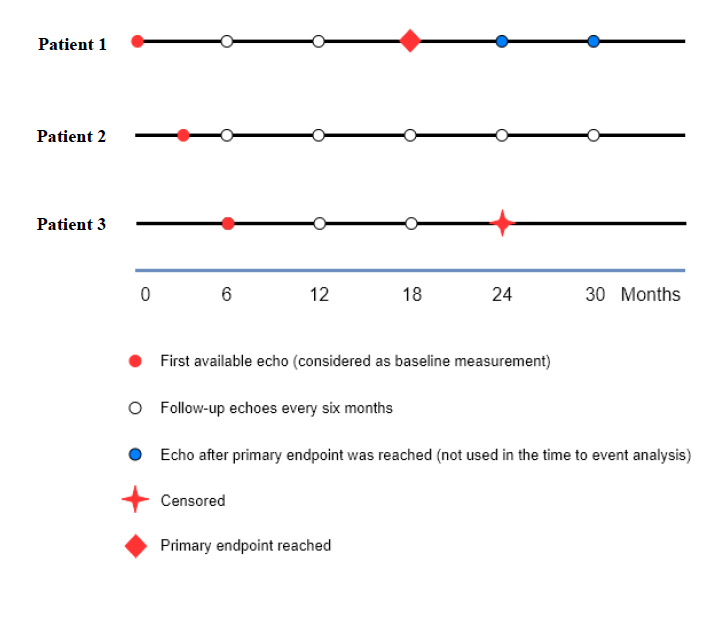


**Supplementary figure 1: Study design: first available and follow-up echocardiograms**

The figure provides 3 example patients to illustrate which echocardiograms were the first available echocardiograms, considered as ‘baseline’ in the analysis (red circles), and at which time-points follow-up echocardiograms were scheduled. 55% of the first available echocardiograms were performed at baseline (follow-up time zero), 12.8% were performed during the first study follow-up visit (target follow-up time 3 months) and 18% were performed during the second follow-up visit (target 6 months). Subsequently, echoes were performed every six months.

**
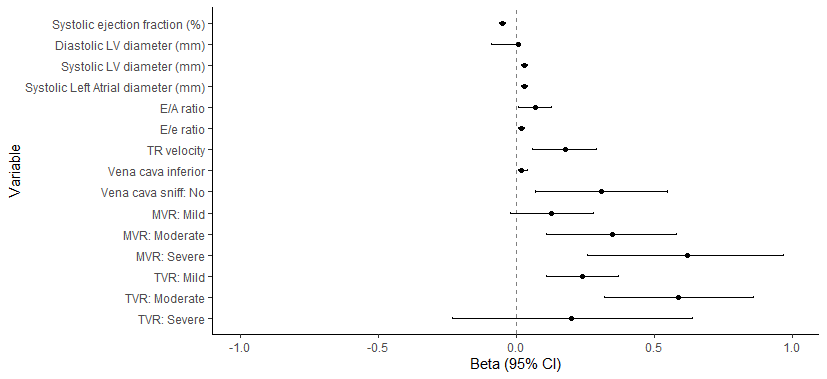
 Supplementary figure 2: Associations of serially measured echocardiographic parameters with serially measured GLS.**

Betas depict change in GLS (in %) when the explanatory variable is increased by 1 unit. 95%CI: 95% confidence interval. MVR: Mitral valve regurgitation. TVR: Tricuspid valve regurgitation)


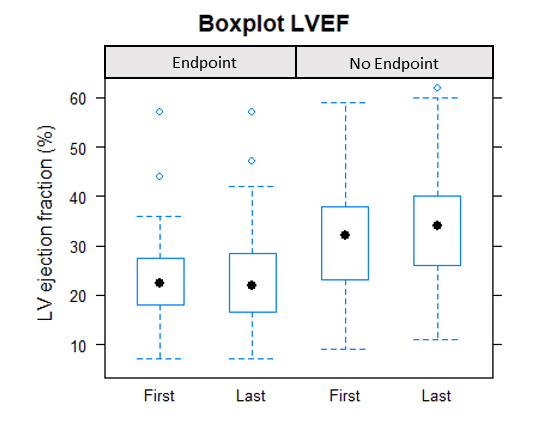

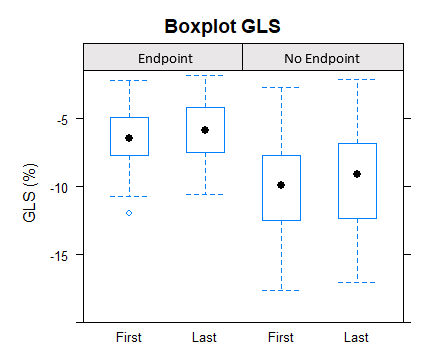


**Supplementary figure 3: First and last GLS and LVEF values according to endpoint status.**

The boxplots show the average GLS and LVEF at the first and last available measurements. The averages of GLS and LVEF in patients with the endpoint is shown in the left panel, whereas the average GLS in patients without the endpoint is shown in the right panel.


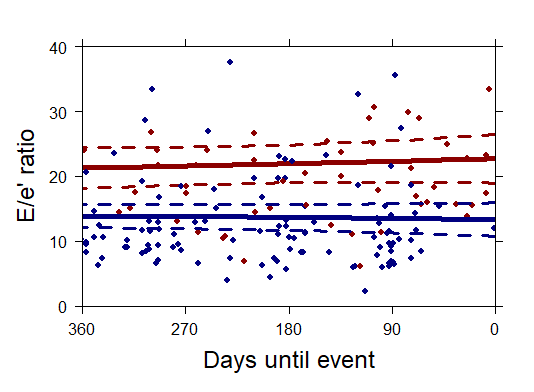

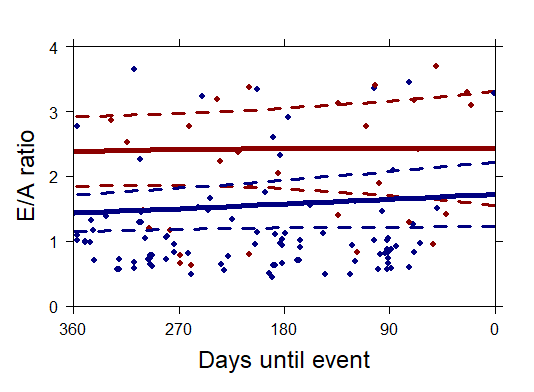


**Supplementary Figure 4: Mean temporal patterns of E/e’ and E/A ratio until occurrence of the primary endpoint or censoring.**

Continuous lines represent mean temporal patterns for patients with the PEP (red) and patients who remained PEP-free (blue), as extracted from the joint model. Time-point zero represents the occurrence of an event in the PEP patients and censoring in patients who remained PEP-free. Dotted lines represent 95% confidence intervals. Each dot represents a single measurement.


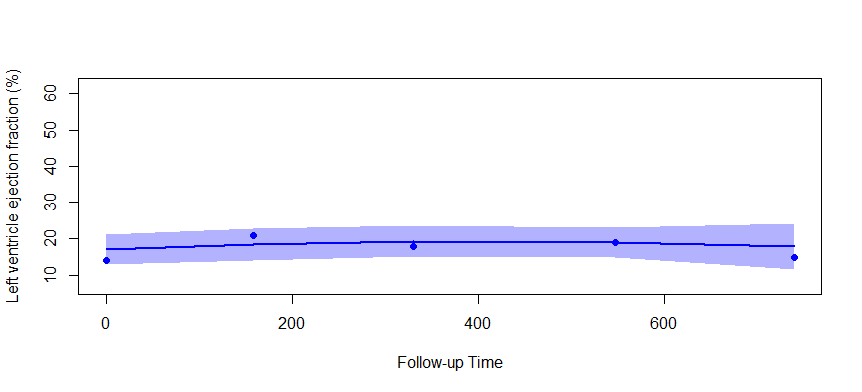


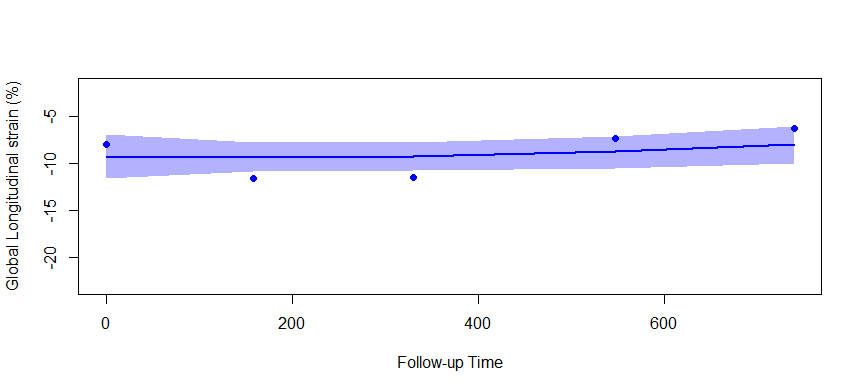


**Supplementary Figure 5: Temporal evolution LVEF and GLS for one example patient.**
